# Increased Proliferation of Neuroblastoma Cells under Fructose Metabolism Can Be Measured by Isothermal Microcalorimetry

**DOI:** 10.3390/children8090784

**Published:** 2021-09-07

**Authors:** Nicola Pini, Zihe Huo, Stefan Holland-Cunz, Stephanie J. Gros

**Affiliations:** 1 Department of Pediatric Surgery, University Children’s Hospital Basel, Spitalstr. 33, 4031 Basel, Switzerland; nicolanpini@gmail.com (N.P.); zihe.huo@ukbb.ch (Z.H.); stefan.holland-cunz@ukbb.ch (S.H.-C.); 2 Department of Clinical Research, University of Basel, Schanzenstrasse 55, 4021 Basel, Switzerland

**Keywords:** thermogenesis, glycolysis, tumor cell proliferation, hypoxia, low nutrient supply, tumor progression, anaerobic metabolism

## Abstract

Neuroblastoma, like other cancer types, has an increased need for energy. This results in an increased thermogenic profile of the cells. How tumor cells optimize their energy efficiency has been discussed since Warburg described the fact that tumor cells prefer an anaerobic to an aerobic metabolism in the 1920s. An important question is how far the energy efficiency is influenced by the substrate. The aim of this study was to investigate how the metabolic activity of neuroblastoma cells is stimulated by addition of glucose or fructose to the medium and if this can be measured accurately by using isothermal microcalorimetry. Proliferation of Kelly and SH-EP Tet-21/N cells was determined in normal medium, in fructose-enriched, in glucose-enriched and in a fructose/glucose-enriched environment. Heat development of cells was measured by isothermal microcalorimetry. The addition of fructose, glucose or both to the medium led to increases in the metabolic activity of the cells, resulting in increased proliferation under the influence of fructose. These changes were reflected in an enhanced thermogenic profile, mirroring the results of the proliferation assay. The tested neuroblastoma cells prefer fructose metabolism over glucose metabolism, a quality that provides them with a survival benefit under unfavorable low oxygen and low nutrient supply when fructose is available. This can be quantified by measuring thermogenesis.

## 1. Introduction

With its origin in the medulla or the sympathetic paravertebral ganglia, neuroblastoma is the most common solid tumor of sympathetic nerves in childhood. Furthermore, it presents with noticeably different clinical outcomes [1,2]. Most patients are diagnosed at an early age and almost half of them have a poor survival rate of less than 40% [3]. Although in recent years many advancements in diagnosing and treating neuroblastoma patients have been made, there is still much research needed to fully understand and cure this disease. More recent approaches towards understanding this tumor and developing new treatment options focus on the tumor’s microenvironment.

The microenvironment of many solid tumors, including neuroblastoma, is often characterized by unfavorable conditions for cell survival. Tumor cells, however, have undergone adaptive changes to escape the fate brought on to them by low oxygen and low nutrient supply. They can, for example, adapt to hypoxia by transforming their phenotype to a more hypoxia-resistant and often aggressive one. We have previously shown that metastatic cell lines exhibit a higher thermogenic profile than primary tumor cell lines that were derived from the same patient [4]. Furthermore, we were able to evaluate treatment response of patient-derived tumor slice cultures to inhibition of hypoxia-dependent targets by monitoring thermogenic changes [5].

Although energy retrieval from glucose is most efficient under aerobic conditions, it has been established by Otto Warburg almost a century ago that given the choice tumor cells prefer to use anaerobic pathways to metabolize glucose [6]. This, undoubtedly, gives these cells an immense survival benefit. Although glucose is the main glycolytic energy source, it has been shown that tumor cells can sustain life on fructose alone. Moreover, it has been proposed that fructose is enhancing the Warburg effect for cancer growth [7]. We hypothesize that a fructose-rich cell environment enhances energy-dependent cell function and proliferation in neuroblastoma cells.

Thermogenesis of tumor cells is a reflection of all cellular processes. It can be measured by isothermal microcalorimetry [8]. It has been shown that this highly sensitive method can detect even small changes in the lipid metabolism by detecting changes in the thermogenic profile [9]. We hypothesize further that changing the metabolism of tumor cells through supplying the cells with either glucose, fructose, or a combination of both, can lead to changes in the metabolic activity of the cells that can be detected by isothermal microcalorimetry.

The aim of this study was to investigate how the metabolic activity of neuroblastoma cells is stimulated by addition of glucose or fructose to the medium and if this can be detected by using isothermal microcalorimetry.

## 2. Materials and Methods

### 2.1. Cell Proliferation

Neuroblastoma Kelly, (ECACC/ Sigma-Aldrich, Munich, Germany) and SH-EP Tet-21/N cells (reported by Lutz et al. [10,11], kindly provided by G. Eschenburg, Hamburg, Germany) were cultivated in RPMI media containing 10% FCS. If possible, aliquots of early passages (4–6) after purchase or receiving were used for all experiments. All cells were cultured in a humidified atmosphere at 37 °C in air with 5% CO_2_ under normoxic conditions. Cells were diluted to a concentration of 8 × 10^4^ cells/mL in Kelly and 5 × 10^4^ cells/mL in Tet-21/N cells. They then were cultivated in 24-well plates containing either 440 µL media, media with added fructose, glucose or both, all at a final concentration of 25 mM. Cell count was performed after a time of 6, 12 and 24 h and viability was determined.

### 2.2. Isothermal Microcalorimetry

A 48-channel isothermal microcalorimeter (calScreener, Symcel AB, Stockholm, Sweden) was used for microcalorimetric measurements as we have previously described [12]. Neuroblastoma cells were seeded into measuring vials and were allowed to attach for 6 h in RPMI medium in the incubator. Glucose, fructose and glucose/fructose were added to the vial to a final concentration of 25 mM each. The sealed vials were then inserted in the well-plate microcalorimeter as suggested by the company. An inert sample was included as a reference. To optimize performance several reference vessels containing medium were added and could be used as a thermal reference. Following thermal equilibration measurements were recorded with the thermostat set at 37 °C. Microcalorimetry data were recorded at a frequency of 1 data point every 60 s over >250 h until the baseline of the metabolic heat signal was reached. Data were saved by the Symcel calView software, which allows export of data as a CVS file. Data were analyzed using GraphPad Prism 8.4 Software. The assay was performed in quadruplicate. A two-sided *t*-test or analysis of variance was applied for testing of significance.

## 3. Results

**Figure 1:**
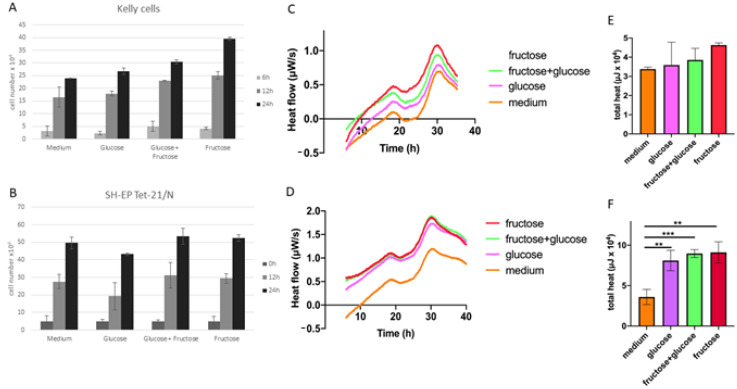
(**A**,**B**) show proliferation of Kelly and SH-EP Tet-21/N cells, respectively, in medium, glucose-enriched, fructose/glucose-enriched and glucose-enriched medium over time. Proliferation is increased after addition of fructose, especially in Kelly cells. SH-EP Tet-21/N cells do not show this effect so clearly. However, the overall proliferation level in SH-EP Tet-21/N cells is higher under the influence of fructose and fructose/glucose compared to glucose and medium alone. (**C**,**D**) present the thermogenic curve of Kelly and SH-EP Tet-21/N cells, respectively, in medium, glucose-enriched, fructose/glucose-enriched and glucose-enriched medium as heat flow (µW/s) over time (h). Fructose-treated Kelly cells clearly show an increased heat flow compared with glucose- and fructose/glucose-treated cells or cells in medium alone. SH-EP Tet-21/N cells show increased heat flow in both fructose and fructose/glucose-treated cells, closely followed by glucose alone (** *p* < 0.01 compared to medium). SH-EP Tet-21/N cells grown in medium alone show lower thermogenic activity. (**E**,**F**) show the overall heat of treated cells in total heat (µJ × 10^4^) over the observation period. SH-EP Tet-21/N cells show significantly increased overall heat, to the greatest extent in fructose (** *p* < 0.01 compared to medium) and fructose/glucose-treated cells (*** *p* < 0.001 compared to medium), followed by glucose alone (** *p* < 0.01 compared to medium). The overall heat mirrors the heat flow.

We investigated proliferation changes of neuroblastoma cell lines under the influence of glucose, fructose and a combination of both. Our assessment revealed that adding glucose to the medium led to an increased proliferation after 12 h and 24 h in Kelly cells compared to using medium alone (Figure 1A). An increase of proliferation from glucose to glucose–fructose was observed but not significant. The effect of increased proliferation was accentuated even more by adding fructose alone. However, this effect was not as pronounced in SH-EP Tet-21/N cells. Although the combination of glucose and fructose as well as adding fructose alone led to a slight increase of proliferation compared with medium alone after 12 h and 24 h, the same concentration did not lead to an increased proliferation in cells grown with added glucose alone (Figure 1B). The results of this seemingly simple assay clearly show that providing tumor cells with different sugars to stimulate metabolism leads to marked changes in tumor cell behaviors. Although these proliferation changes were reproducible and conclusive, differences were statistically not significant. This led us to consider if these even slight changes in cell metabolism were of biological relevance. 

In order to investigate the metabolism of Kelly and SH-EP Tet-21/N cells we performed isothermal microcalorimetry. We analyzed the thermogenic profile (heat flow (µW/s) over time (h)) of cells that were grown in regular medium, medium with added glucose, glucose/fructose and fructose in the same concentration as in the proliferation assay as heat flow (µW/s) over time (h). Thermogenic curves show a general increase of metabolic activity over the first 30 h in both cell lines. Kelly cells show the highest activity in the fructose group, followed by the glucose/fructose group and the glucose group compared with the cells grown in medium alone, which show the least thermogenic activity (Figure 1C). SH-EP Tet-21/N show the highest activity in cells stimulated with fructose and glucose/fructose, closely followed by glucose alone while medium alone contrary to the proliferation assay shows much less thermogenic activity (Figure 1D). When analyzing the overall heat production as total heat (µJ × 10^4^) for the observation time of the four groups, these findings were confirmed. In Kelly cells, adding fructose led to the greatest overall heat production, while in SH-EP Tet-21/N cells the fructose and glucose/fructose group produce increased heat to a similar extent, while the medium alone and glucose group produce less (Figure 1E,F).

Overall, the microcalorimetric results mirror the results of the proliferation assay with the exception of the SH-EP Tet-21/N grown in medium alone. This might be due to that fact that not only proliferation but also other cellular processes enhanced by sugar contribute to the increased thermogenic profile. Moreover, even small changes in the cells metabolic activity induced by adding different sugars led to changes in proliferation and activity of the cells. These changes are strong enough to be detected by changes in their thermogenic profile.

## 4. Discussion

### 4.1. Thermogenesis

Thermogenesis can highly sensitively be measured by isothermal microcalorimetry of cancer cells [4]. It can be detected in the range of microwatts under isothermal conditions [8]. Heat production is a reflection of all cellular processes for which energy is necessary. The metabolism of tumor cells, for example, is significantly elevated compared with the metabolism of other cells. By this highly sensitive method, it could be shown that small changes in the lipid metabolism led to changes in the thermogenic profile [9]. There are several other applications for isothermal microcalorimetry such as microbiological drug testing, monitoring in material testing and in food microbiology, as well as in parasitological research [12,13,14,15]. We have successfully deployed isothermal microcalorimetry in the timely testing of drug response in a rare and highly malignant case of pediatric clear cell sarcoma of the kidneys [5]. Moreover, we recently published a manuscript describing a higher thermogenic profile for metastatic tumor cells in matching cell lines originating from metastatic lymph nodes compared to primary tumor of the same patient. Metastatic cells were less adhesive compared with primary tumor cells [4]. Our present results show that isothermal microcalorimetry is a sensitive method to detect slight changes in tumor cell thermogenesis, which can be the result of cellular changes in tumor cells metabolism through stimulation with glucose or fructose, as in our case, or of otherwise induced changes of cell properties.

### 4.2. Tumor Cell Metabolism

Cancer cells have a greater need for energy than non-cancerous cells; they grow more rapidly and have an unlimited cell division potential. In the 1920s, Otto Warburg found that instead of oxidative phosphorylation cancer cells prefer to produce adenosine triphosphate (ATP) by glycolysis, which is a less efficient pathway in terms of energy retrieval [6]. Anaerobic metabolism of fructose via fructokinase to fructose-1-phosphate, via aldolase to dihydroxyacetone phosphate, and via triose-phosphate isomerase to glycerinaldehyde-3-phoshate entering glycolysis is more energy efficient than metabolism of glucose to glycerinaldehyde-3-phoshate. Saving ATP by using this pathway and metabolizing fructose enables cellular mechanisms to work more efficiently. In addition, the presence of fructose-carrying transport systems vs. transport systems carrying glucose only will influence the pathway taken by the cell. Our results show that both neuroblastoma cell lines prefer metabolizing fructose to glucose. While Kelly cells prefer fructose alone SH-EP Tet-21/N cells utilize fructose and a fructose/glucose mixture similarly effectively.

Glucose is one key glycolytic substrate for cancer cells. It serves as an energy source as well as facilitator of the anabolic production of metabolites such as amino acids, nucleotides and fatty acids [16,17,18]. Clinically, the enhanced glucose metabolism of tumor cells can be utilized to monitor the course of the disease by using positron emission tomography (PET) which depicts an enhanced cellular uptake of [18F]-FDG (2-deoxy-2-[18F]-fluoro-D-glucose). Unfortunately, FDG-PET imaging only detects certain tumor types and is neither completely specific nor sensitive for tumor detection [19]. It was hypothesized that one possible explanation for this could lie in the fact that little more than half of human malignomas express one of the major glucose transporters GLUT1 [20]. Peng et al. showed that inhibition of GLUT1 in neuroblastoma cells led to a decrease in cell proliferation, inducing cell cycle arrest [21]. It has been suggested that inhibition of GLUT1 could present a target for tumor therapy [21,22]. Furthermore, the inactivation of GLUT1 leads to a reduced proliferation, invasion and migration of the neuroblastoma cells [21].

The primary fructose transporter GLUT5 is expressed on the cell surface of various tumor cell types, including but not limited to colorectal carcinoma and human breast cancer [23,24]. Fructose metabolism increases aerobic glycolysis and down-regulates mitochondrial respiration [25]. Fructose intake has been associated with cancer growth and has been shown to be up-regulated in various cancers [23,24]. Over-expression of GLUT5 might accelerate this process even further. The ability of cells to adapt to fructose metabolism constitutes a survival benefit of tumor cells under low oxygen supply and thus enhance metastases [26]. Glioma cells, for example, can utilize fructose via GLUT5 in a glucose-free medium and survive [27]. Another advantage of the fructose metabolism is that the cell can utilize fructose even under low oxygen conditions. This in turn can accelerate glucose metabolism and produce metabolites such as lactate [28].

The role of glucose and fructose metabolism has also been the focus of several studies on neuroblastoma. Neuroblastoma can be characterized by a hypoxic microenvironment within the tumor. It is hypothesized that the ability to survive this hostile environment is driven by the adaptation of the tumor cells. This includes adaptation to low oxygen and low nutrient supply, amongst others. Fructokinase, an enzyme of great importance in the fructose metabolism by making it possible to bypass the glycolytic regulatory steps in glycolysis, is regulated by HIF-1α, a master regulator of hypoxia [29]. Cells that can survive tumor hypoxia and metastasize have most probably undergone a selection process, which enables them to withstand an unfavorable environment with low oxygen and poor nutrient supply. Being able to metabolize fructose gives neuroblastoma cells the advantage to perform and survive under these unfavorable conditions. One possible explanation for the increase of thermogenic activity and proliferation of Kelly neuroblastoma cells in fructose-rich medium compared with addition of only glucose or glucose-fructose in our experiment could be that these cells have already undergone a selection process towards these unfavorable conditions and thrive under fructose.

### 4.3. Tumor Cells Proliferation and Migration

In breast cancer cells it has been shown that the presence of fructose increases the migratory potential and proliferation of the cells compared with the presence of glucose [30]. The mechanisms for this remain unclear. One hypothesis is that variations in the oligosaccharide structures contribute to migration by altered folding, stability, or biologic function of glycoproteins on the cell surface [31]. Tumor-specific glycan structure changes, for example, can modify cell matrix adhesion and migration via modulation of integrin clustering or related signal transduction pathways [32]. Our results confirm that the addition of glucose, and even more relevantly of fructose increase proliferation of neuroblastoma cells. Moreover, the stimulated cells show a higher thermogenic potential. It has been hypothesized that metastasizing cells have a higher thermogenic profile than non-migrating cells [9]. Therefore, as Monzavi-Karbassi et al. [30]. described for breast cancer cells, a stimulation of neuroblastoma cells with added glucose or, in our case, even more potently with fructose, might not only result in a higher thermogenic profile but also in an increased metastatic potential.

### 4.4. Clinical Significance

With growing evidence for severe metabolic differences between different cell populations of heterogeneous tumors such as neuroblastoma and between primary and metastatic tumor cells, tumor metabolism is becoming an increasingly important marker for malignancy. Measuring the metabolic activity of patient tumor tissue or cells with a reliable and fast method such as isothermal microcalorimetry will enable us to create thermogenic profiles for different tumor entities and distinguish between cells with a higher and lower metastatic potential in the future.

## 5. Conclusions

The investigated neuroblastoma cell lines prefer fructose metabolism over glucose metabolism, a quality that provides them with a survival benefit under unfavorable low oxygen and low nutrient supply. This can be quantified by measuring thermogenesis.

## Data Availability

All data is presented in the manuscript.
